# Microtome-integrated microscope system for high sensitivity tracking of in-resin fluorescence in blocks and ultrathin sections for correlative microscopy

**DOI:** 10.1038/s41598-017-13348-6

**Published:** 2017-10-19

**Authors:** Nicolas Lemercier, Volker Middel, Didier Hentsch, Serge Taubert, Masanari Takamiya, Tanja Beil, Jean-Luc Vonesch, Tilo Baumbach, Patrick Schultz, Claude Antony, Uwe Strähle

**Affiliations:** 1 Institut de Génétique et de Biologie Moléculaire et Cellulaire, 1, rue Laurent Fries, 67404 Illkirch, France; 2 Centre National de la Recherche Scientifique, UMR7104, 1, rue Laurent Fries, 67404 Illkirch, France; 3 Institut National de la Santé et de la Recherche Médicale, U964, 1, rue Laurent Fries, 67404 Illkirch, France; 4 Université de Strasbourg, 1, rue Laurent Fries, 67404 Illkirch, France; 5 Institute of Toxicology and Genetics, Karlsruhe Institute of Technology, Hermann-von-Helmholtz-Platz 1, 76344 Eggenstein-Leopoldshafen, Germany; 6 Laboratory for Applications of Synchrotron Radiation, Karlsruhe Institute of Technology, Kaiserstr. 12, 76131 Karlsruhe, Germany

## Abstract

Many areas of biological research demand the combined use of different imaging modalities to cover a wide range of magnifications and measurements or to place fluorescent patterns into an ultrastructural context. A technically difficult problem is the efficient specimen transfer between different imaging modalities without losing the coordinates of the regions-of-interest (ROI). Here, we report a new and highly sensitive integrated system that combines a custom designed microscope with an ultramicrotome for in-resin-fluorescence detection in blocks, ribbons and sections on EM-grids. Although operating with long-distance lenses, this system achieves a very high light sensitivity. Our instrumental set-up and operating workflow are designed to investigate rare events in large tissue volumes. Applications range from studies of individual immune, stem and cancer cells to the investigation of non-uniform subcellular processes. As a use case, we present the ultrastructure of a single membrane repair patch on a muscle fiber in intact muscle in a whole animal context.

## Introduction

A key challenge in biomedicine and biotechnology is to link the function of genes from their phenotypic effects in an organ and tissue context to the molecular events occurring at the subcellular level. Imaging is a central technology to understand these processes in space and time. The coverage of the necessary wide ranges of spatial and temporal resolution requires the combination of different imaging modalities. Fluorescence microscopy allows imaging of biological specimens with high resolution offering also measurement of real-time kinetic data in living systems. A disadvantage of fluorescence microscopy is that the investigator remains blind to the non-fluorescent cellular context including elements like filaments, vesicles, membranes, and organelles^1,2^. For high resolution imaging of these cellular structures, electron microscopy and tomography are the imaging modalities of choice. In other instances, two light based modalities such as, for example, confocal microscopy and super resolution fluorescence microscopies have to be combined^3–7^. In many instances, switching from one modality to another entails the processing of the specimen such as fixation, embedding in resin, sectioning, staining etc. A particular critical issue is the tracking of the ROI without losing its coordinates during the processing and transfer. When rare or unique events in intact tissue or organisms are to be investigated, an additional complication is presented by the large tissue volume in which the ROI has to be found.

In recent years, huge progress has been made to tackle the challenge of correlating light and electron microscopy (CLEM) imaging^1,8–11^. However, these protocols are anything but routine yet^3,6,12–16^. Issues are specimen fixation and processing for electron microscopy and their effects on the preservation of fluorescent signal or cellular detail^3,14,16–18^. The challenge posed by “rare” events or structures that are represented by single or a few cells only in a large piece of tissue calls for further developments^1^. Examples are metastasizing cancer cells, stem cells, macrophages in their natural tissue context or a single myofiber with a membrane lesion in an intact muscle. Due to the narrow field of view of high-resolution imaging methods, a prerequisite to find such “rare” structures is the trimming of the tissue block down to the ROI accompanied by efficient tracking methods in the subsequent processing of the sections cut from the tissue block.

Here, we report the construction of a new instrument that overcomes many of the above mentioned technical hurdles and greatly facilitates the tracking and capture of scarce structures/events. A compact and highly sensitive epifluorescence microscope was developed and mounted onto an ultramicrotome to perform both fluorescence tracking and imaging on a single instrumental setup and at each step of the specimen preparation protocol. While an another set-up was reported recently^11^, our Microtome-Integrated-Microscope (MIM) is characterized by a remarkable sensitivity of in-resin detection of fluorescence by employing a new design of a lIght weight microscope optimized for photon collection. By having integrated this microscope directly onto the ultramicrotome, one can follow the ROI at each step from specimen localization in the block, block trimming, sectioning, tracking sections with fluorescence to documentation of landmarks and relative coordinates of the ROI. We have developed methods to assess the precise 3D location of the ROI in the block reducing sectioning efforts enormously. The MIM is highly suitable for detecting weak fluorescence signal or localizing rare events in large tissue pieces or small organisms.

We applied our new method to study the ultrastructure of the membrane repair patch after muscle membrane wounding in the intact muscle. Repair proteins and lipids rapidly accumulate at the site of wounding forming a tight patch re-establishing the integrity of the barrier to the extracellular space^19,20^. In skeletal muscles of zebrafish embryos, this repair patch is removed by macrophages as a prerequisite for the complete restoration of the lipid bilayer of the sarcolemma^19^. The damaged plasma membrane has previously been subjected to ultrastructural analysis in tissue culture cells and sea urchin eggs^21,22^. However, none of the previous studies focused on the repair of an individual damaged cell *in situ* in the tissue context of a healthy animal at the ultrastructural level. We show that the repair patch *in vivo* consists of an amorphous mass and displays various vesicle profiles and membrane stretches. Moreover, for the first time, we show here ultrastructural details of a macrophage interacting with a membrane repair patch. These user cases demonstrate that the MIM is optimally suited to reliably detect single cellular and subcellular structures in large tissue volumes or in intact zebrafish embryos.

## Results

### Design features of the microtome-integrated microscope (MIM)

**Figure 1:**
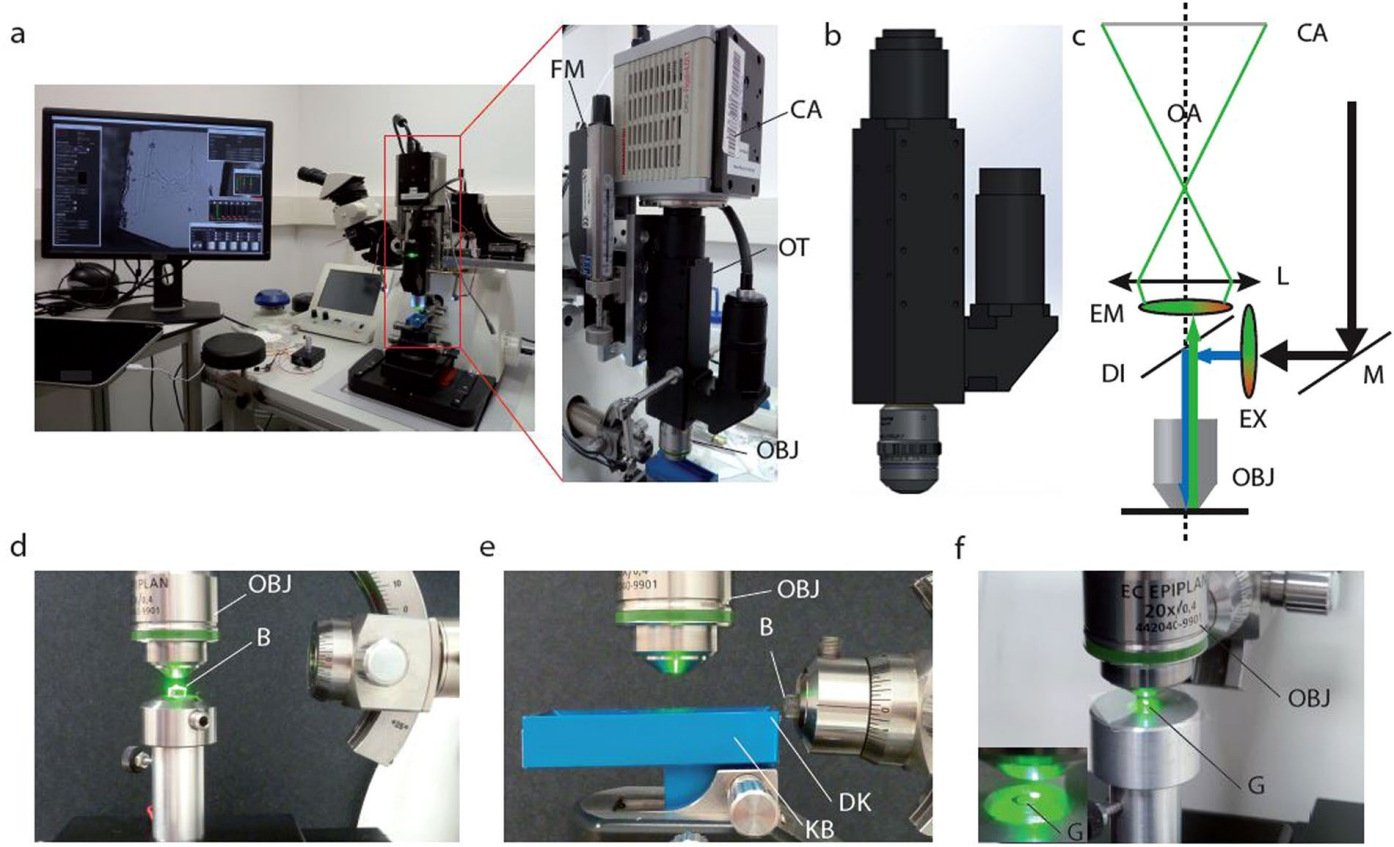
Microtome-Integrated Microscope (MIM). (**a**) The MIM is directly mounted on an UC7 Leica ultramicrotome and connected to the detector. The MIM and stereomicroscope are positioned at a right angle. The objective is facing the boat of the Jumbo knife to scan the ribbons for fluorescence detection. The MIM is composed of a high-sensitivity sCMOS Hamamatsu ORCA-Flash4.0 LT Camera (CA) installed on a customized optical tube tube (OT) equipped with a plan apochromatic lens and a dual-band fluorescence filter cube. The MIM can use various objective lenses (OBJ) as detailed in Table 1. (**b**) Model view of the MIM showing the structure of the optic tube with air objective and the connection to the fluorescence source on the side. (**c**) Optical scheme of the MIM showing the air objective (OBJ), the excitation (EX) and emission (EM) filters separated by the dichroic mirror (DI). Following the optical axis (OA), the light rays are focused on the sCMOS detector (CA) by a planapochromatic lens (L). The fluorescence source is reflected on the excitation filter (EX) by a mirror (M). (**d**) Side view of the MIM during sample processing, showing block-face imaging with the objective lens (OBJ) facing the Block (B) in the microtome sample holder. (**e**) Floating ribbon scanning with the objective lens above the knife boat (KB) during sectioning of the block (B) and with the diamond knife (DK). (**f**) “On-grid”- section imaging (G), also visible in inset with the same objective lens (OBJ).

It is critical that fluorescence signals can be detected in the 3D space of the tissue block before and during sectioning. To this end, the MIM was mounted on the head of an ultramicrotome (Leica, UC7) perpendicular to a stereomicroscope (Fig. 1a–c). This configuration allows switching from trimming and cutting under the stereomicroscope to image acquisition with the MIM by simply rotating the microtome head.

**Table 1:** Specifications of the objectives used on the MIM.

	Magnificsation	Numerical aperture	Working distance (mm)	Field of view	Pixel Size (µm)	Calculated resolution (nm)	Measured resolution (nm)^1^
Leica HC PL Fluotar	5x	0.15	12	2.6 mm × 2.6 mm	1,28	2480	
Leica HCX PL Fluotar	10x	0.3	11	1.3 mm × 1.3 mm	0,65	1240	
Zeiss EC Epiplan	20x	0.4	4.1	540 µm × 540 µm	0,32		
Leica N Plan L	20x	0.4	3.2	540 µm × 540 µm	0,32	930	
Leitz Plan L	40x	0.6	3.5	340 µm × 340 µm	0,16	620	978
Leica HCX PL Fluotar	100x	0.75	4.7	135 µm × 135 µm	0,063	496	

^1^Lateral resolution was measured with 100 nm fluorescent beads, 610 nm light on the basis of a 2D PSF.

The MIM was designed to image the sample through air with long working distance objectives to avoid physical contact with the fragile sections. To detect faint fluorescence signals even deep in the tissue block, we optimized the MIM for light sensitivity: (1) A short phototube was built with a minimum of lenses and (2) connected to an ultra-sensitive camera (sCMOS) (Fig. 1a–c). The optical tube was equipped with a plan apochromatic lens (diameter, 22 mm; focus 200 mm) to be used with standard infinity corrected objective lenses. Moreover, the tube lens was corrected for three colors facilitating the detection of overlapping fluorescent signals of different colors.

The MIM has a low weight and is thus easily moved via a 3-axis stage over a large area to examine the resin block, the sections floating in the boat or the sections transferred onto EM grids. The microscope’s position in Z is set by a motorized and computer-controlled focussing actuator (Newport SMC-100CC) with micrometer accuracy to stably preserve the focus during image acquisition and user handling (Fig. 1). The setup is controlled by custom software (MIM Manager) written in C, Python and OpenCL (Supplementary Fig. 1). The MIM manager offers open and integrated solutions for fluorescence settings (Supplementary Fig. 1d), focussing control (Supplementary Fig. 1b,c) and image acquisition in the same graphical user interface (Supplementary Fig. 1a). The Hamamatsu camera C++ SDK library has been wrapped in Python2.7 using ctypes allowing expert users to access buffer and all camera functionalities. A camera manager is also available for less experienced users to control live image capture parameters (Supplementary Fig. 1e). By using the camera manager and its implemented movie mode, several frames of the ROI can be averaged on the GPU or the CPU by default. This led to a dramatically increased signal-to-noise ratio specifically helping to detect very weak fluorescence patterns. Large fields of view can be recorded at high magnification by tiling of individual frames. These composite images are useful to find the ROI in large tissue areas. To facilitate image alignment in the electron microscope, landmarks were documented with the MIM relative to the ROI on the sections (Supplementary Fig. 3). The same reference points need to be identified in the transmission electron microscope (TEM) (Supplementary Fig. 3b) and, with the help of the coordinate converter GUI (Supplementary Fig. 3e), an affine transfer matrix between the two coordinate systems including scaling, rotation and translation factors were calculated (Supplementary Fig. 3a,d). These parameters are then applied to determine the precise location of the ROI in the TEM (Supplementary Fig. 3c). The images can be saved in 8 or 16 bit (native mode) or 32 bit data array in five different formats (Tiff, Jpeg, PNG, Spider, MRC) for processing the coordinate conversion from the MIM setup to the compustage of the electron microscope (Supplementary Fig. 1).

We developed a method called Depth Evaluation of Axial Position (DEAP) to measure the distance of the ROI from the block surface (Supplementary Fig. 2). A plain copper grid was deposited on the block surface with a micromanipulator (Supplementary Fig. 2a). The MIM was focused on the grid surface illuminated from above with reflected white light (Supplementary Fig. 2b). The grid was removed and the MIM was then focused on the fluorescent ROI (Supplementary Fig. 2c). The difference between these two positions was then calculated by taking into account both grid thickness and the refractive index of Lowicryl HM20 to compensate for the axial focus shift (Supplementary Fig. 2d–f). We measured positions of ROIs at least 200 μm deep in the resin block with an accuracy of 1 to 2 μm. By employing the DEAP method, we reduce sectioning and subsequent scanning of ribbons and sections significantly.

### The optical properties of the MIM

As assessed with a glass lamella micrometer, the magnification of the optical tube (M_tube_ = 10) of the MIM and its camera is close to that of a standard epifluorescence microscope. The total magnification (M_total_) is given by the product of the magnification of the objective (M_obj_) and the optical tube (M_tube_ = 10). The overall magnification ranges from 50- to 1000-fold. The MIM provides working distances between 3.2 and 12 mm and a field of view between 2.6 mm × 2.6 mm to 135 μm × 135 μm with a pixel size from 1.28 to 0.063 μm (Table 1).

**Figure 2:**
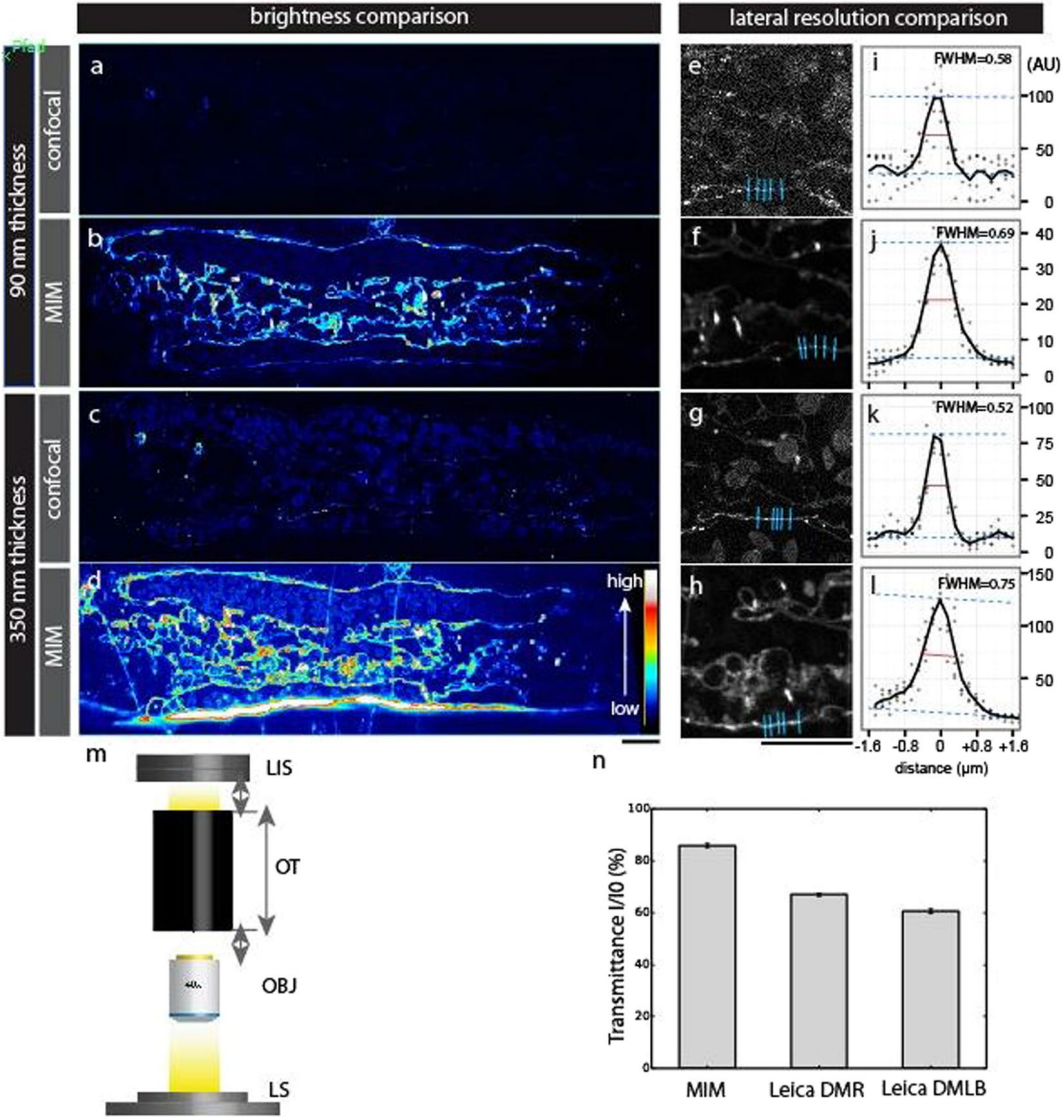
Evaluation of the optical features of the MIM relative to commercial microscopes. (**a–d**) Ultrathin 90 nm (**a**,**b**) and semi-thin 350 nm (**c**,**d**) Lowicryl sections from a high-pressure-frozen 3-day old embryo expressing *Tg(kdrl:Hsa.Hras-mCherry)* in blood vessels. Fluorescence images from a Leica SP5 confocal microscope (**a**,**c**, merge of 81 images of the same field of view; 63x, NA 1.2 objective with 3x zoom) and MIM **(b**,**d**, 40x, NA 0.6 objective) were shown in a color-code to visualize fluorescence signal intensity. For comparison with MIM, we optimized carefully the parameters of confocal imaging to have the very best image quality by using a high precision HCX PL APO CS 63.0 × 1.20 WATER UV objective: laser intensity (561 nm at AOTF 20%), PMT gain (869 V), emission detection range (583–662 nm), pixel dwell time (2.5 µsec), pinhole size (1 airy unit), averaging (2), and pixel size (80 nm). Sections recorded with MIM and confocal microscope represent images of adjacent parasagittal sections cut from the same animal. Scale bar: 10 µm. (**e**–**l**) Lateral resolution comparison between confocal and MIM. Magnified views of a-d for caudal vein wall are shown in (**e**–**h**), respectively. Levels of fluorescence intensities were normalized to facilitate comparison. Scale bar: 10 µm. Fluorescence profiles (arbitrary unit [AU]) along the line drawn perpendicular to a vein endothelium wall (distance = 0) toward both directions (+/−1.6 µm) were plotted for each condition (n = 5 juxtapositions, blue lines in e-**h**). Full width at half maximum (FWHM, red line) of vein endothelium wall for each condition was measured. (**m**) Exploded view of the light transmission measurement set-up. The transmitted light intensity through inner light pathway lenses and prisms of the optical tubes (OT) was assessed with the same objectives (OBJ) and light source (LS) for the MIM and two commercial epifluorescence compound microscopes. The camera was replaced by a light intensity sensor (LIS) on the tested instruments. (**n**) The transmitted light intensity is represented for MIM and the two tested epifluorescence microscopes (Leica DMR, Leica DMLB). The MIM performs better in light detection than the two tested commercial epifluorescence microscopes.

We first assessed the performance of the MIM by comparing it with a standard confocal microscope (Leica SP5). We chose a confocal microscope as reference since confocal imaging is the method to detect fluorescence signals deep in the tissue of our application model, the intact zebrafish embryo. We cut 90 and 350 nm thick parasagittal sections through a 3-day old zebrafish embryo expressing membrane-tagged mCherry (*Tg(kdrl:Hsa.HRAS-mCherry*) in blood vessels^23^. For comparison, we imaged the adjacent sections from the same transgenic embryo under a confocal microscope. The MIM yielded brighter signals from sections of the same thickness than the confocal microscope (Fig. 2a–d). When using the vessel walls as reference, the lateral resolution of the MIM is comparable to that acquired in the confocal system with 80 nm pixel size (Fig. 2e–l; for details of the settings of the confocal microscope, see legend of Fig. 2). Yet, the confocal system under the given settings requires approximately four times thicker sections to match the same signal-to-noise levels of the MIM.

To further assess the ability of the MIM to image weak fluorescent signals, we measured the light transmission of the MIM phototube in comparison to two standard epifluorescence wide field microscopes. We replaced the cameras by a light intensity sensor and used the same light source and objective on each of the three tested instruments (Fig. 2m). The light transmission of the MIM phototube (85%) is higher than that of standard epifluorescence microscopes (67% and 61% for Leica DMR and Leica DML, respectively) (Fig. 2n).

We calculated the theoretical lateral resolutions of the different lenses used on the MIM (Table 1). In addition, we measured the lateral resolution for the 40x air objective (NA 0.6) using 100 nm fluorescent beads and 610 nm excitation light. Based on a 2D PSF, the lateral resolution of the MIM is 979.8 nm under these conditions. The resolution across the field of view and vignetting effects are dependent on the objective rather than the phototube itself. Since the MIM employs commercially available plan apochromate objectives (Table 1) vignetting effects should be marginal. In agreement, we did not observe vignetting effects with a apochromate lens and also the field of view was homogenous (Fig. S5). In summary, the improved light transmission properties together with the integrated sCMOS camera system make the MIM a highly sensitive device to detect fluorescent signals in resin-embedded tissues and cells.

### Multi-step MIM workflow for CLEM

**Figure 3:**
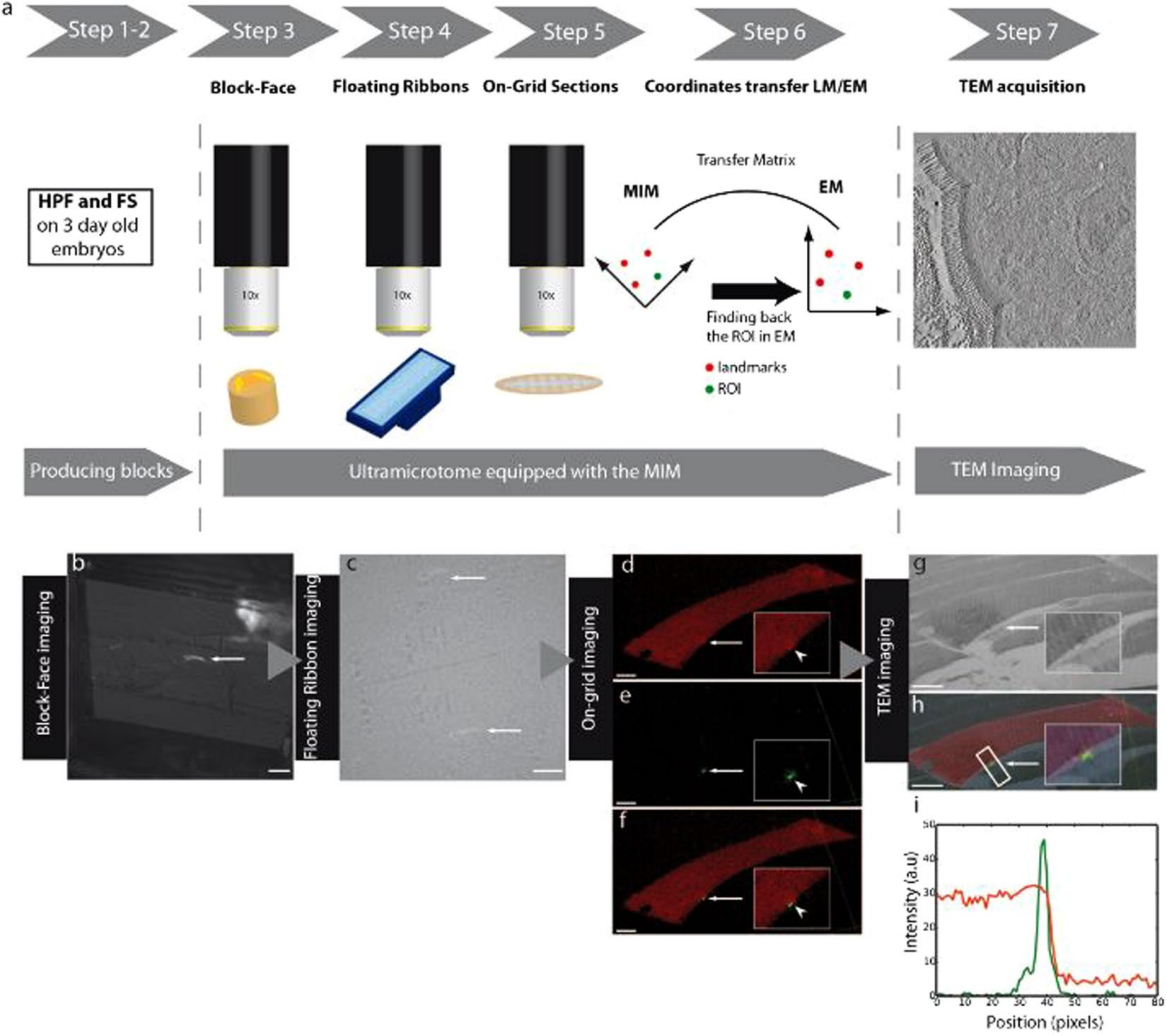
Fluorescence tracking of membrane repair in zebrafish embryos using the MIM. (**a**) Workflow for fluorescence tracking of the ROI from sample cryofixation (HPF, step 1) and freeze-substitution (FS, step 2) to multi-step fluorescence tracking under the MIM including whole-embryo block-face imaging (step 3), fluorescence tracking on floating serial sections (step 4) and higher resolution « on-grid » imaging (step 5) and recording of coordinates of the ROI (step 6) followed by transmission electron microscopy (TEM) or tomography imaging (step 7). (**b**) Block-face fluorescence tracking of a single membrane injured myofiber (arrow) prior to trimming (10x objective). Scale bar, 100 µm. (**c**) Fluorescence tracking on floating serial sections immobilized with hair filaments on the boat of a Jumbo diamond knife (Diatome) acquired with the 10x objective (arrows). Scale bar, 100 µm. (**d–f**) Detailed fluorescence pictures of the cell of interest in 260 nm sections (40x objective) in pseudocolour after transfer to formvar-coated EM-slot-grid. Scale bar, 10 µm (5 µm in insets). The region pointed out by the arrow (arrow head in inset) of panel d corresponds to the lesion site. Note accumulation of Dysf-mCherry in the repair patch is not strongly visible due to the high gain in order to outline the entire myofiber. The lesioned region is recognizable in the red channel as a bump-like, local distortion of the cell boundary (see also higher magnification in the inset of panel (d). The green dot (**e**, arrow) corresponds to AnxA2-mEosFP accumulation in the repair patch. (**f**) Merged views of panels d and e show unequivocally that the chosen myofiber is the single myofiber in the embryo that has been damaged with the laser. (**g**) EM low magnification (450x) image of the same lesioned cell showing the position of the lesion superimposed in (**h**) with the fluorescence picture (arrow). Scale bar, 10 µm. The fluorescence intensity profile (**i**) across the myofiber (white rectangle (**h**)) highlights the localization of the green dot in the membrane.

The MIM allowed us to develop an innovative CLEM strategy from cryofixation to electron microscopy (Fig. 3a). This concise workflow referred to as “Direct-CLEM” considerably reduces sample handling as compared with other CLEM protocols. Moreover, it offers high flexibility in connection with other CLEM strategies such as grid scanning with immersion objectives^3,16^, block-face imaging in a SEM^12,24^, TEM, electron tomography^8,12,16,25^ or super-resolution imaging^3,6,7^.

As an example, we chose the ultrastructural analysis of a membrane repair patch present on a single myofiber in intact zebrafish muscle. Individual myofibers were tagged with fluorescent membrane repair proteins AnxA2-mEosFP (green) and Dysf-mCherry (red) and a membrane lesion was inflicted by pulses with an infrared laser as previously described^19,20^. The trunk/tail regions of the embryos with the injured myofibers were cryo-immobilized by high pressure freezing. By choosing vitrification as method of tissue fixation, we reached a close-to-native ultrastructure and excellent preservation of fluorescence^3,16,26^. The fluorescence of both mCherry and mEosFP were well maintained in the Direct-CLEM workflow.

**Figure 4:**
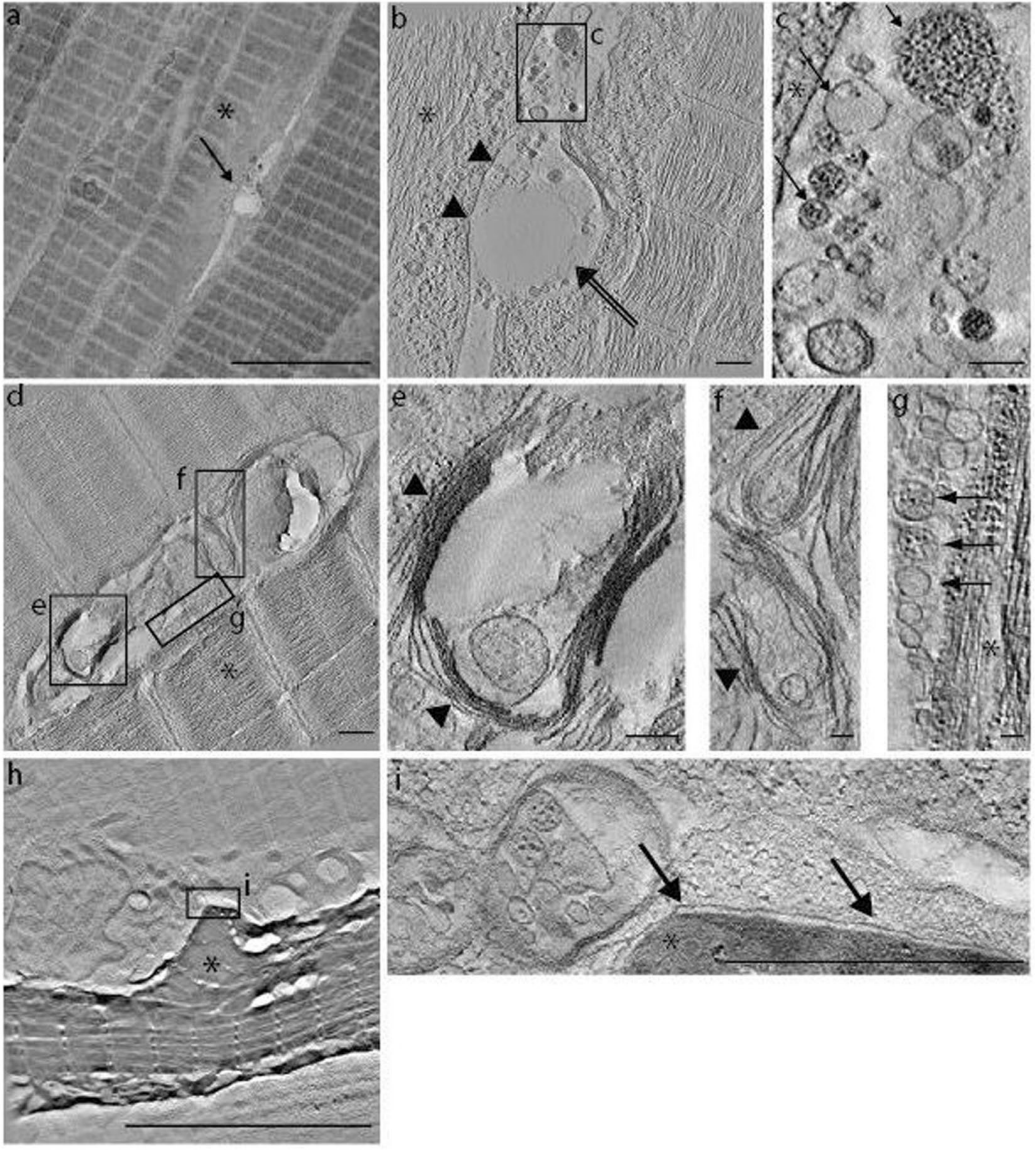
High resolution imaging of the lesion patch by electron tomography. (**a**) Overview of muscle tissue with a membrane repair patch between two muscle fibers (arrow). Scale bar, 10 µm. (**b**) Tomographic slice through the repair patch in panel (**a**) at 9600x magnification. The sarcolemma of the lesioned cell (asterisk) is already resealed (black arrowheads). Note that, the sarcomere organization appears still disturbed. A characteristic, rather thick and slightly electron dense coat, is lining the sarcolemma extracellularly over the lesioned region (double black arrow) while the lower right myofiber seems also affected by the laser. Scale bar, 500 nm. (**c**) The lesion site shows heterogeneous vesicle profiles with a grainy or a smooth aspect (black arrow in c). Scale bar, 200 nm. (**d**) Tomographic slice of a large repair patch at 9600x magnification with heterogeneous membranous profiles shown in detail in panels (**e**,**f**,**g**). Membranes appear loosely stacked in a multilamellar configuration (black arrowheads in **e**,**f**). Scale bar, d, 500 nm; (**e**), 200 nm; (**f**), 100 nm; (**g**), 100 nm. (**g**) Some vesicles of various sizes are also visible forming a row against the lesioned myofiber. These vesicles also show heterogeneity in their aspect (black arrows in **g**) as noticed in (**c**). (**h**) Image of a macrophage sitting on the repair patch. The plasma membrane of the macrophage shows a close apposition with the repair patch (black arrow in **i**). Scale bar, **h**, 10 µm; (**i**), 1 µm. The damaged cell shows a stronger contrast in comparison to the surrounding undamaged cells and appears shrunk. This presumably reflects that the sarcolemma damage triggered an apoptotic program in the myofiber as previously described for cells with large lesions (Middel *et al*., 2016).

After “clean-cutting” the surface of the resin block, the depth of the ROI in the block was determined with micrometer precision using the DEAP method. Predetermining the depth of the ROI with the DEAP method allowed rapid trimming of the block down to the depth of the ROI (Fig. 3b) followed by careful sectioning and collection of serial sections from the selected volume containing the ROI. For electron tomography, 250–260 nm thick sections were cut and ribbons of serial sections floating in the diamond boat were directly scanned for the presence of the repair patch using the 10x and 40x objectives (Fig. 3c). After transfer of positive sections onto formvar-coated EM-slot grids, the identity of the cell with the repair patch was unequivocally confirmed by the detection of both red (Dysf-mCherry, entire myofiber) and green (AnxA2a-mEosFP, repair patch) (Fig. 3d–f,i). Images of the fluorescent repair patch and of three reference points were then acquired with the 20x objective to record coordinates for detection of the ROI in the electron microscope (Supplementary Fig. 3c). Tilt series were acquired and tomograms of the repair patch at the wounded site were calculated (Fig. 3g,h, Fig. 4, Supplementary Movie 1). Taken together, this workflow illustrates the strength of the MIM which allows carrying out all necessary preparative and imaging steps with one instrument hence minimizing the risk to damage the fragile sections.

### Ultrastructure of the membrane repair patch in a single muscle fiber of an intact zebrafish embryo

As a test case of the MIM and its associated workflow, we determined the ultrastructure of membrane repair patches in otherwise intact muscle tissue. Membrane lesions were introduced into individual myofibers of the somitic muscle of zebrafish embryos, and the repair patches were fluorescently tagged as described in the previous section^19,20^. The Direct-CLEM workflow (Fig. 3a) was applied and the ultrastructure of six repair patches was reconstructed by electron tomography. The lesion patch from different injured cells did not have a uniform structure (Fig. 4a,b,d). From patch to patch varying membranous vesicular (Fig. 4c,g) and lamellar structures (Fig. 4e,f) of different size were embedded in an amorphous mass (Fig. 4b,d). The stacked membrane structures observed are reminiscent of multilamellar bodies (Fig. 4e,f; Supplementary Movie 1, Supplementary Fig. 4). We previously showed that macrophages interact preferably with the repair patch of the damaged cells^19^. The plasma membrane of the macrophage sitting next to the damaged cell (Fig. 4h) is tightly apposed to the repair patch forming a very tight interaction (Fig. 4i). Large vesicles reside close to the plasma membrane of the macrophage (Fig. 4h,i).

Taken together, these examples provide new insights into plasma membrane repair in an intact tissue context that have not been visualized at an ultrastructural level before and would not have been possible without the MIM. The MIM provides an unprecedented way to reliably find and image such “rare” and discrete structures embedded in a large tissue volume.

## Discussion

Many areas of biological research demand the combined use of different imaging modalities for example to cover a wide range of magnifications or to record complementary information such as placing fluorescent patterns into a high resolution ultrastructural context. A technically difficult and largely unsolved problem in CLEM is the transfer of the same specimen between different imaging modalities without losing the ROI^14,25,27^. Here, we report a new and highly sensitive microtome-integrated imaging system that combines a purpose-designed microscope mounted on a commercial ultramicrotome with a workflow (Direct-CLEM) optimized for efficient selection of plastic sections comprising the ROI.

The optical components of the MIM were reduced to the minimum to optimize gain of photons. This optimized phototube achieves a light detection that is higher than the tested commercially available, epifluorescent widefield microscopes. A key feature of the MIM is the possibility to perform with one compact instrument all operations from imaging of the blockface, trimming of the block to tracking fluorescent signals in sections floating in the boat as ribbons or residing individually on EM-grids. This is facilitated by imaging through air avoiding transfer of the fragile sections between instruments or use of immersion lenses. In return, the associated lower numerical apertures of the air lenses of the MIM limit image resolution slightly relative to that of commercial epifluorescence microscopes operated with immersion lenses used previously to detect in-resin fluorescence^3,14^. However, this is not a major concern for the performance of the MIM and the Direct-CLEM method as the ease of handling and the light sensitivity are more critical for efficient signal tracking, section preparation and documentation.

The light weight of the MIM together with its in-build control system (MIM Manager) allows rapid shuttling from one observation position to the next, thus improving handling and throughput. The unique instrumental specifications of the MIM help to significantly reduce the number of sections to be analyzed. Especially with the DEAP method, the location of ROIs can accurately be estimated deep in the resin block thereby providing a reliable trimming guide to limit the number of sections to be cut and scanned. Taken together, the MIM permits to track in-resin fluorescence signal at every step of the “Direct-CLEM” workflow without imprinting external references on the block surface or the EM-grid^28,29^. The “direct-CLEM” is both a preparative and an analytic workflow. New CLEM developments focus on automated FIB-SEM analysis which is very efficient but less discriminative for a weakly fluorescent spot of interest^7,11^. Moreover, the MIM and the associated “Direct CLEM” provides an effective correlative bridge between many different modalities such as widefield and confocal fluorescence microscopies and higher power technologies such as super-resolution^3,6,7^, X-ray^30,31^ and TEM^6,12,16,32^.

Depending on the chosen objective lenses, the MIM offers a large field of view which can even be increased by stitching adjacent images together. The limitation of the size of tissue to be scanned is not imposed by the optical constraints of the MIM but by the volume of tissue that can be effectively vitrified by high pressure freezing. Previous reports on correlated methods lack the sensitivity of detection^26^ or require immersion objectives^14^ to detect in-resin fluorescent signals. In a recent publication, locator tools for tracking fluorescence in resin-embedded cells via blockface imaging with a custom-built fluorescence mini-microscope have been described^11^. The ultraLM and miniLM locator tools are very efficient to operate in an automated way in TEM or SEM to find the positive cells of interest, The MIM, on the contrary, with its sensitivity-optimizing strategy is too sensitive to be automated as one needs to keep full control of the instrument while imaging in order to avoid false positives as we look for very fine fluorescence features. Moreover, MIM and Direct-CLEM allow detection and superimposition of multiple fluorescent signals.

Our new instrumental set-up together with the developed “Direct-CLEM” workflow is particularly suitable to investigate rare or unique structures in large tissue volumes by correlative techniques. Areas of potential applications at the tissue or cell population level range from studies of individual immune cells, stem cells to metastasizing cancer cells in a native tissue context and include studies of non-uniform or rare cellular and subcellular processes. The MIM/Direct-CLEM should be of great interests for any researcher who wishes to track fluorescently labeled events in a tissue or a small organism. Applications range from fields like biomedicine, developmental biology, molecular and cellular biology to biotechnology. A typical example of such “rare” events is our presented case of the repair of a membrane lesion in the sarcolemma of a muscle fiber of the intact muscle^33^.

Up to now, the membrane repair processes have not been studied in the native tissue context of an intact, healthy muscle at the ultrastructural level. Our data show that the repair patch is tightly squeezed between the injured and the neighboring cells. Moreover, the injured cell retained tight contact with the neighboring uninjured muscle tissue. In all examples examined, the patch formed at the lesion contained amorphous material. In addition, the patch enclosed varying filamentous and membranous structures. The membranous profiles detected in the repair patch are heterogeneous in size and form with varying locations in the different repair patches examined. They may be accumulating material for resealing of the plasma membrane. Another interpretation is that these structures represent membranous debris entrapped by the precipitating repair proteins at the damaged area. It remains to be elucidated whether the amorphous regions of the patches have a structural organization as previously suggested by the ordered arrival of different repair proteins at the lesion site^20^. Detailed immunogold labeling experiments^34,35^ are needed to understand whether the repair patches contain structured assemblies of the different repair proteins.

Clearly the plasma membrane of the upper left myofiber in Fig. 4a has been restored again excluding the repair patch from its cytoplasm. This cell shows still extensive damage of the sarcomeres below the newly formed sarcolemma. Thus, the membrane of damaged myofibers seems to be repaired on the cell-proximal side of the patch. By real-time confocal imaging, we recently demonstrated that macrophages are required to remove the repair patch^19^. The repair patch accumulates phosphatidylserine in a dysferlin-dependent manner. The latter serves as an “eat-me” signal for macrophages^19^. The previous confocal real-time analysis showed that the macrophage takes up the repair patch in smaller portions. At the ultrastructural level, the macrophage is tightly aligned along the repair patch. This tight association of the macrophage may help maintaining the integrity of the barrier function while the macrophage removes pieces from the repair patch. The cytoplasm of the macrophage next to the repair patch was rich in various sized vesicles. These vesicles may be involved in phagocytosis of the repair patch.

Addressing these open questions in detail requires more comprehensive studies far beyond the proof-of-principle experiments presented here. The presented examples underscore, however, clearly the power of the MIM and the associated Direct-CLEM workflow. Detection of structures such as an individual repair patch in a large tissue space would not have been possible without the MIM.

## Methods

### Zebrafish husbandry

Zebrafish husbandry and experimental procedures were performed in accordance with German animal protection regulations (Regierungspräsidium Karlsruhe, Germany, AZ35-9185.81/G-137/10). Publication of protocols and machines used was approved by the IGBMC authorities. *Tg(kdrl:Hsa.HRAS-mCherry)* ^*s916*^ was used to visualize blood vessels^36^.

### Cloning, microinjection and myofiber damage

Cloning was carried out following standard procedures (supplementary information). Muscle expression of fusion constructs was driven by the unc45b promoter^37^. Approximately 100 eggs were used for each injection. The eggs were injected (DNA concentration of 20–80 ng/µl) between 1–2 cell stage with a drop of ~1/10^th^ the size of the yolk using a glass injection needle. At 3–5 dpf embryos were embedded into 0.5% low melting point agarose to immobilize them onto a microscopy glass slide (detailed protocol see supplementary information). Muscle membrane lesions were introduced into single expressing muscle fibers as described previously^19^.

### Cryofixation, freeze substitution and plastic embedding

Zebrafish embryos were cryofixed (vitrified) 15–30 min post membrane damage by high pressure freezing. The embryo was cut due to limitations of the carrier size and only the posterior half was high pressure frozen. After freeze substitution the samples were embedded into Lowicryl HM20 resin and blocks were polymerized using UV light (for detailed protocol see supplementary information).

### Construction of the MIM

The MIM was constructed using the mechanical and optical workshops of the IGBMC. The MIM will be built by the IGBMC workshops for interested parties upon request. Interested laboratories can directly contact Didier Hentsch or Nicolas Lemercier.

### Trimming and sectioning

Serial sections (250–260 nm thick) were prepared from the blocks using an UC7 ultramicrotome (Leica, Austria) equipped with a Jumbo knife (Jumbo histo diamond knife 45°, Diatome, Switzerland) with a very large diamond boat hence facilitating collection and arrangement of the serial sections.

For the localization of the ROI in the block we used a method called Depth Evaluation of Axial Position of ROI (DEAP) to measure the distance of the ROI from the block surface (Fig. S2). To this end, a copper plain grid (50 µm single hole copper grids; Electron Microscopy Science, GA50-Cu) was deposited on the block surface with a micromanipulator (Fig. S2a) and the MIM was focused on the grid surface illuminated by reflected white light (Fig. S2b). The grid is then removed and the MIM is focused on the fluorescent ROI (Fig. S2c). The difference between these two positions is then calculated by taking into account both the grid thickness and the axial focus shift induced by the Lowicryl refractive index (Fig. S2d–f). With this information, the excess resin from the surface to slightly above the ROI can be removed safely in large steps followed by serial sectioning in a controlled way through the ROI.

Long ribbons were carefully collected and parked for immobilization in stretches perpendicularly to the diamond-boat with hair-filaments arranged in a ladder-like pattern^38^. The immobilized ribbons were scanned with the 10x objective of the MIM. Once the positive sections were identified, they were transferred onto EM-slot grids (2 × 1 mm slot formvar-coated EM grids; Agar Scientific, G2500PD). Once the sections of interest were secured onto EM grids, they were observed at higher magnification with the 40× objective of the MIM and checked for the presence of a red dot (dysferlin accumulation at the lesion site) and green dot (accumulation of Annexin A2a) at the same site, and images were acquired. The coordinates of the lesion spot as well as those of additional three reference points were recorded.

### TEM and electron tomography

The sections stuck on EM grids were contrasted using uranyl acetate (4% (w/v) in 70% methanol) for 2 min, then after rinsing with 50% methanol in water, they were further stained with Reynolds lead citrate for 1 min^39^. Tomography data were acquired on a FEI Tecnai FEG30, operating at 300 kV with a CMOS camera (One View, Gatan 4 Kx4K, Gatan Inc. USA) or on a FEI Tecnai FEG20, operating at 200 kV equipped with a CCD Ultrascan camera (Gatan 2 Kx2K, Gatan Inc. USA). In both cases, the tilt series were acquired with 1° increment from −60° to +60°, single axis^38^, using SerialEM^40^ acquisition software, and reconstructed using etomo^41^. To find the lesion site in the tomography microscope, we calculated the affine transfer matrix between the MIM and the TEM coordinate system, including scaling, rotation and translation factors (Fig. S3a,d). To find these parameters we tracked the three reference points in both LM and EM (Fig. S3b). The LM coordinates were recorded using Fiji or ImageJ software. The matrix was then used to calculate the TEM coordinates of the ROI (Fig. S3c,d). A coordinate converter GUI (Fig. S3e) allowed the users to enter the coordinates of the selected points and performed automatically the required calculation in a “user-friendly” way (Fig. S3e). The motorised stage then moved the grid to the right place (ROI) for tilt series acquisition.

### Open source protection

#### Creative-Commons-MIM

The whole MIM assembly, the DEAP method and related devices described in the manuscript, the control software and the associated workflow are licensed under Creative Commons Attribution Non-Commercial Share-Alike license (CC-BY-NC-SA).

## Electronic supplementary material

Supplementary information

Supplementary Video
