# Supplementary material for: Microtome-integrated microscope system for high sensitivity tracking of in-resin fluorescence in blocks and ultrathin sections for correlative microscopy
Source: Sci Rep. 2017 Oct 19;7:13583. doi: 10.1038/s41598-017-13348-6 (PMC5648784; doi:10.1038/s41598-017-13348-6)
Supplement: Supplementary file 1 — Supplementary information [file 41598_2017_13348_MOESM1_ESM.pdf]

Supplementary material

Microtome-integrated microscope system for high sensitivity tracking of in-resin fluorescence in blocks and ultrathin sections for correlative microscopy

Nicolas Lemercier[1,2,3,4,7], Volker Middel[5], Didier Hentsch[1,2,3,4], Serge Taubert[1,2,3,4], Masanari Takamiya[5], Tanja Beil[5], Jean-Luc Vonesch[1,2,3,4], Tilo Baumbach[6], Patrick Schultz[1,2,3,4,7], Claude Antony[1,2,3,4,7] and Uwe Strähle[5,7]

Online methods

Zebrafish husbandry

Zebrafish husbandry and experimental procedures were performed in accordance with German animal protection regulations (Regierungspräsidium Karlsruhe, Germany, AZ35-9185.81/G-137/10). *Tg(kdrl:Hsa.HRAS-mCherry)*^{s916} was used to visualize blood vessels[1].

Cloning

All cloning of tagged repair proteins was carried out following standard procedures[2] and instructions of suppliers of reagents as indicated. Primers for cloning (Supplementary Table 1) were purchased from Metabion (Germany, Planegg). PCR to amplify small inserts (<700 bp) was performed using GoTaq or GoTaq2 polymerase (Promega, Germany, Mannheim or Fermentas, Germany, Darmstadt), whereas longer PCR products were amplified with Pfu polymerase (Promega, Germany, Mannheim) using a gradient-PCR protocol (50°C - 70°C annealing temperature). The elongation time was chosen according to the insert length (GoTaq: 1000 bp/min; Pfu: 500 bp/min). PCR products were separated on an ethidium bromide/agarose gel, excised and purified with a gel purification kit (Peqlab, Germany, Erlangen) followed by restriction enzyme digestion of inserts and backbone vectors (NEB, Germany, Frankfurt am Main and Fermentas, Germany, Darmstadt) at 37°C for 1 h. After digestion, vectors and inserts were again gel purified as described above. The vectors were incubated with thermosensitive alkaline phosphatase (Fermentas, Germany, Darmstadt) for 1 h at 37°C and again gel purified. Ligation was set at a ratio of 3:1 insert to vector and incubated at 16°C over night with T4 DNA Ligase (Promega, Germany, Mannheim) followed by transformation into E.coli XL1blue or Top10. Expression vectors for fusion proteins were cloned with the Gateway system and LR clonase II kit (Invitrogen, Germany, Darmstadt). The unc45b promoter (from -505 to -310 relative to the ATG of *unc45b*) was subcloned into the 5' entry vector (p5E) between BamHI and SacII sites for muscle specific expression[3]. All cDNAs fused either N- or C-terminally, spaced by a linker sequence to fluorescent proteins were subcloned into the middle entry vector (pME). For C-terminal fusion constructs, mEosFP[4, 5] or mCherry[6] were PCR amplified and inserted in the MCS of the pME vector between SpeI and SacI sites. For N-terminal fusion, mEosFP was inserted between KpnI and XhoI sites, whereas for mCherry, XhoI and ClaI sites were used. A

linker sequence (3x[Gly-Gly-Gly-Gly-Ser]) was used to provide flexible spacer between the proteins-of interest and the fluorescent protein. For C-terminal fusion, the linker sequence was integrated into the primer, whereas for N-terminal fusion, the linker was separately inserted into the pME backbone using XhoI and ClaI. cDNAs were cloned to fuse with fluorescent proteins N- or C-terminally according to published data[7]. The truncated version of zebrafish (ClaI/SpeI) dysferlin were tagged C-terminally. CAAX was amplified and fused N-terminally to mEosFP between ClaI/SpeI sites. AnxA2a was amplified and fused C-terminally to mEosFP between KpnI and SpeI sites.

All plasmids were sequenced and verified. p5E and pME vectors were transformed into XL1blue E.coli and plated on LB + kanamycin plates. The desired promoter in the p5E, the fusion protein in the pME, the p3E-polyA (Tol2kit #302) and the pDEST (Tol2kit #394) were recombined according to the manual of the LR clonase II kit (Invitrogen, Germany, Darmstadt). The reaction was incubated over night at room temperature. Top10 E.coli were transformed and plated on LB + ampicillin plates. Plasmid DNA was prepared following the instructions of the supplier of the reagents (Qiagen, Germany, Hilden).

Microinjection

The collected zebrafish eggs were cleaned and approximately 100 eggs were used for each injection. The eggs were transferred onto a 10 cm petridish lid and water was removed. Embryos were injected (Eppendorf FemtoJet) through the chorion directly into the yolk during the 1-2 cell stage. Injections were carried out with purified plasmid DNA (20-80 ng/µl; midiprep, Qiagen) diluted in H_2O supplemented with 0.01% phenol red. One nl (~1/10 of the yolk size) was injected per embryo. Injection needles were pulled from borosilicate glass capillary tubes with filament (0.58 mm diameter, Warner Instruments) using a micropipette puller (Sutter Instruments).

Preparation of embryos for laser wounding of sarcolemma and imaging

Injected embryos were grown to 3-5 dpf at 28°C. 3- to 5-day-old larvae were prepared as previously published[7]. By using a hair-forceps (a hair is taped to a 100 µl pipette tip), the embryos were embedded in 0.5% low melting point agarose supplemented with 0.02% MESAB and positioned onto a microscopy glass slide. Embryos were strictly oriented with the head to the left and tail to the right (dorsal up, ventral down).

By using a confocal microscope (TCS SP2, Leica Microsystems, Mannheim, Germany), the sarcolemma of single cells co-expressing AnxA2a-mEosFP and a C-terminal fragment of Dysf[8] fused to mCherry were chosen and damaged at 32x zoom using HCX APO L 63x/0.90 W U-V-I CS2 objective with a Ti:Sa two-photon laser (MaiTai, Spectra Physics, Mountain View, CA) set to 860 nm (gain 65%, offset 26%) using the region-of-interest (ROI) set to 0.25-2 µm². Two scans (4x line alignment) were performed. Following minigene injection into the zygote, the repair proteins are expressed in a mosaic fashion in the muscle cells. Cells dorsal to the tip of the yolk extension were preferentially chosen for inflicting laser damage to their membranes. Tiling pictures were taken of the damaged cell and the surrounding undamaged repair protein expressing cells as landmarks with the confocal microscope (1x zoom) to find the damaged cells once embryos were embedded in resin blocks.

Cryofixation, freeze substitution and plastic embedding

For optimal structural preservation of the embryos in a close-to-native state, the treated embryos were cryofixed (vitrified) using a Compact-02 High Pressure Freezer (Wohlwend Cie, Senwald, Switzerland) within about 15-30 min after the damage had been inflicted[9]. The embryos were rinsed in 20% polyvinylpyrrolidone (PVP 10.000, Sigma Aldrich, ref #101380720) in E3 medium and only the posterior half of the embryo was placed in the sample carriers (3 mm diameter; 150 µm depth, Wohlwend Cie, Senwald, Switzerland). The latter are frozen in PVP under 2100 bar with liquid nitrogen[10]. The samples were oriented into sample carriers in the same way as during laser–injury under the two-photon confocal microscope.

The samples were then processed for freeze substitution (FS) in an AFS2 device (Leica, Austria). FS was performed in 0.1% uranyl acetate in pure acetone at -90°C for 12 h[11]. Samples were then rinsed in pure acetone, then in 100% ethanol at -45°C prior to progressive resin infiltration with Lowicryl HM20, (Polyscience) at increasing concentration in ethanol (30%, 50%, 75%, 100%) over 16 h at -45°C. Block polymerization occurred using the AFS2 robot (FSP) under UV illumination for 15 h from -30°C to 0°C, then for another 15 h from 0°C to 20°C.

Supplementary table 1

Primer	Sequence (5' to 3' direction)	Comments
**Fusion vectors**		
F.LinkCspeI	GCGACTAGTGCCGGTGGCGGAGGGTCTGGAG	used to clone the Linker for N- or C-terminal fusion into the pME backbone
R.LinkCnotI	TTGCGGCCGCGGCTGATCCACCCCCGCCG	
F.LinkNxhoI	GCGCTCGAGGCCGGTGGCGGAGGGTCTGGAG	
R.LinkNclaI	TTATCGATGGCTGATCCACCCCCGCCG	
F.CherryC.Link.SpeI	CGAACTAGTGGCGGTGGCGGAGGGTCTGGAG GGGGCGGTTCCGGCGGGGGTGGATCAGTGAG CAAGGGCGAGGAGGAC	used to make C- and N-terminal fusion constructs for mCherry into the pME backbone
R.CherryC.SacI	GCGGAGCTCGCCTTACTTGTACAGCTCGTCCAT G	
F.CherryN.XhoI	CGACTCGAGGGCGCCACCATGGTGAGCAAGG GCGAGGAG	
R.CherryN.Link.ClaI	GCGATCGATGCCTGATCCACCCCCGCCGGAAC CGCCCCCTCCAGACCCTCCGCCACCCTTGTAC AGCTCGTCCATG	
F.EOSC.Link.SpeI	CGAACTAGTGGCGGTGGCGGAGGGTCTGGAG GGGGCGGTTCCGGCGGGGGTGGATCAAGTGC GATTAAGCCAGACATGAAGATC	used to make C- and N-terminal fusion constructs for mEOS into the pME backbone
R.EOSC.SacI	GCGGAGCTCGCCTTACCGTCTGGCATTGTCAG GCAATC	
F.EOSNkpnI	GCCAGGTACCCCGCCACCATGAGTGCGATTAA GCCAGACATG	
R.EOSNxhoI	GCCACTCGAGGGCCCGTCTGGCATTGTCAGG	
**cloning of cDNAs**		
F.shTM-DysfC.ClaI	GCGATCGATGCCACCATGTGGCTTATTCTGGG CCTCTTTATAC	used to clone shortTM-DomainC of zebrafish dysferlin into the pME backbone
R.shTM-DysfC.SpeI	GCCACTAGTGGCCTGTGTTCCCTTTCCTAG	
F.unc45b195BamHI	CAGAGGATCCGCGCTTAATGGTTTCTTACAGTA	used to subclone unc45b.195 promoter into the p5E backbone
R.unc45b195SacII	GCTGCCGCGGGCGATAGGGTCTATTTATGGAG	
F.CAAXEOSNClaI	GCCAATCGATGCCAAGCTGAACCCTCCTGATGA	used to fuse CAAX to mEOS N-terminally into the pME backbone
R.CAAXEOSNSpeI	GCGACTAGTGGCTCAGGAGAGCACACACTTGC	
F.Anxa2a.KpnI	GCGGGTACCGGCGCCACCATGGCTTTGGTCTC TGAATTC	used to fuse AnxA2a to mEOS N-terminally into the pME backbone
R.Anxa2a.SpeI	CGAACTAGTGCCGTCGTCTCCATTGCACAGGC	

Supplemental references

1. Hogan, B.M. et al. Ccbe1 is required for embryonic lymphangiogenesis and venous sprouting. *Nat Genet* **41**, 396-398 (2009).
2. Sambrook, J. & Russell, D.W. The condensed protocols from Molecular cloning : a laboratory manual. (Cold Spring Harbor Laboratory Press, Cold Spring Harbor, N.Y.; 2006).
3. Rudeck, S. et al. A compact unc45b-promoter drives muscle-specific expression in zebrafish and mouse. *Genesis* **54**, 431-438 (2016).
4. Wiedenmann, J. et al. EosFP, a fluorescent marker protein with UV-inducible green-to-red fluorescence conversion. *Proc Natl Acad Sci U S A* **101**, 15905-15910 (2004).
5. Nienhaus, K., Nienhaus, G.U., Wiedenmann, J. & Nar, H. Structural basis for photo-induced protein cleavage and green-to-red conversion of fluorescent protein EosFP. *Proc Natl Acad Sci U S A* **102**, 9156-9159 (2005).
6. Kwan, K.M. et al. The Tol2kit: a multisite gateway-based construction kit for Tol2 transposon transgenesis constructs. *Dev Dyn* **236**, 3088-3099 (2007).

7. Roostalu, U. & Strahle, U. In vivo imaging of molecular interactions at damaged sarcolemma. *Dev Cell* **22**, 515-529 (2012).
8. Middel, V. et al. Dysferlin-mediated phosphatidylserine sorting engages macrophages in sarcolemma repair. *Nat Commun* **7**, 12875 (2016).
9. McDonald, K.L., Morphew, M., Verkade, P. & Muller-Reichert, T. Recent advances in high-pressure freezing: equipment- and specimen-loading methods. *Methods Mol Biol* **369**, 143-173 (2007).
10. Mobius, W. et al. Electron microscopy of the mouse central nervous system. *Methods Cell Biol* **96**, 475-512 (2010).
11. Kukulski, W. et al. Correlated fluorescence and 3D electron microscopy with high sensitivity and spatial precision. *J Cell Biol* **192**, 111-119 (2011).
12. Kremer, J.R., Mastronarde, D.N. & McIntosh, J.R. Computer visualization of three-dimensional image data using IMOD. *J Struct Biol* **116**, 71-76 (1996).

Supplemental figure legends

Figure S1 Microtome Integrated Microscope (MIM) manager control panel

(a) Live image display window. Two modes are available for full resolution and full camera frame view, respectively. (b) Focusing actuator control. (c) Memory focus positions for objective lenses (5x to 100x) can be set by users to rapidly re-identify specific positions. (d) The fluorescence source control window provides seven independent excitation wavelength intensity controls for fluorescence tracking of a wide variety of fluorescent proteins or dyes. (e) The camera manager controls the sCMOS camera for live imaging and capture with various modalities such as montaging or frame averaging and allows saving images in standard EM formats (such as Tiff, MRC, SPIDER).

Figure S2 Depth evaluation of the axial position of ROI (DEAP)

(a) A single hole TEM copper grid (Electron Microscopy Science, GA50-Cu) is placed on the block surface with a 3-axis micromanipulator. (b) The MIM is focused on the grid surface. The block is illuminated by reflection with two white LEDs to detect the grainy texture of the grid. The MIM is moved along the Z-axis until the grainy surface appears perfectly sharp. The thickness of the grid is subtracted to give the exact position of the block surface. This position G is stored as surface reference. (c) The grid is removed and the MIM is focused on the fluorescent ROI. The MIM position E is also stored as estimated ROI focus value

(d) The true ROI axial position in the resin block is deeper than the estimated value due to resin induced refraction. The Lowicryl HM20 has a refractive index (n2=1.5)

higher than air (n1=1). This refractive index mismatch leads to a focus shift. The true position V can thus be calculated by a simple geometric approach based on Snell's law (e) using the angle tangent ratio (f). The difference between the surface position G and the true ROI position V gives the depth of the ROI in the block. The resin can be trimmed with the 45° Diatome trimming knife until the ROI is reached. This procedure dramatically reduces the number of sections to find a single fluorescent structure in the whole resin block.

Figure S3 Coordinate transfer from MIM to EM by affine transformation matrix calculation

(a) The issue is to determine the coordinates of the ROI in the compustage coordinate system of the electron microscope. Finding these values requires to determine the conversion matrix (M) between the two coordinate systems. This matrix contains the scaling, rotation and translation parameters to describe the coordinate system conversion. (b) The matrix is calculated by using three reference points whose coordinates can be easily identified in both light and electron microscope coordinate systems. (c) The matrix parameters are applied to the LM coordinates of the ROI to find their equivalent in EM. The LM coordinates of the reference points are equal to the product of their EM equivalent multiplied by the transfer matrix (d (1)). Determining the conversion matrix consists in multiplying the EM reference points matrix by the inverse matrix of the LM reference points in homogeneous coordinates (d(2)). The parameters are applied to the ROI coordinates as shown in (d(3)). (e) A software has been developed to provide users with a «user-friendly» GUI. After entering the LM ROI coordinates and those from the reference points of LM and EM images, the user first calculates the matrix and then applies the corresponding parameters to release the TEM coordinates of the ROI.

Figure S4 Tomographic 3D model of vesicles and membranes stacks in the repair patch superimposed on a slice of the full volume at 9600x magnification

The model shows the positions of the vesicles through the patch, embedded in the multilayered membrane profiles. The vesicles are distributed at the surface of the lesioned cell.

Figure S5 Effect of objective lens quality on vignetting and field homogeneity of the MIM.

A grid is imaged with a Zeiss epiplan 20x infinity corrected (a) and with a Mitutoyo Plan APO 20X infinity corrected (b). The region in the red square clearly shows the image blurring in the corners with the epiplan objective lens (red arrow in a). With the Plan APO objective, the field is homogeneous (red arrow in b) and the corners are at the same focus as the center of the image.

Movie S1 Tomogram of a repair patch in a lesioned myofiber at 15500x

The corresponding tilt series was acquired on a FEI Tecnai FEG30, operating at 300 kV with a CMOS camera (One View, Gatan 4Kx4K, Gatan Inc. USA) with 1° increment from - 60° to + 60°, single axis, using SerialEM acquisition software. The reconstruction was performed in IMOD using etomo[12]. The tomogram displays the features of several vesicles and membranes in a multilamellar organization through the thickness of the repair patch.

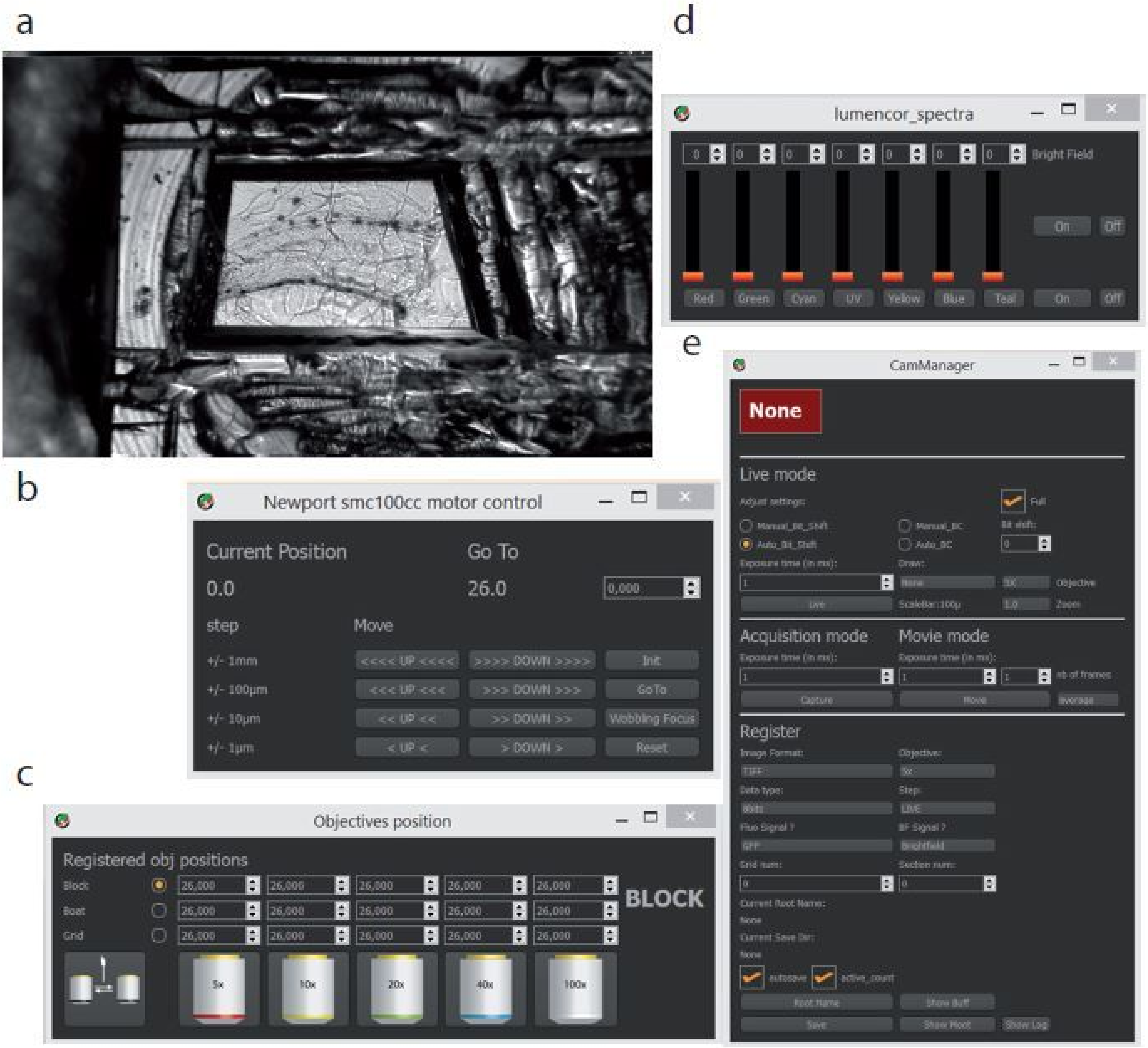

Fig S1 Lemercier et al

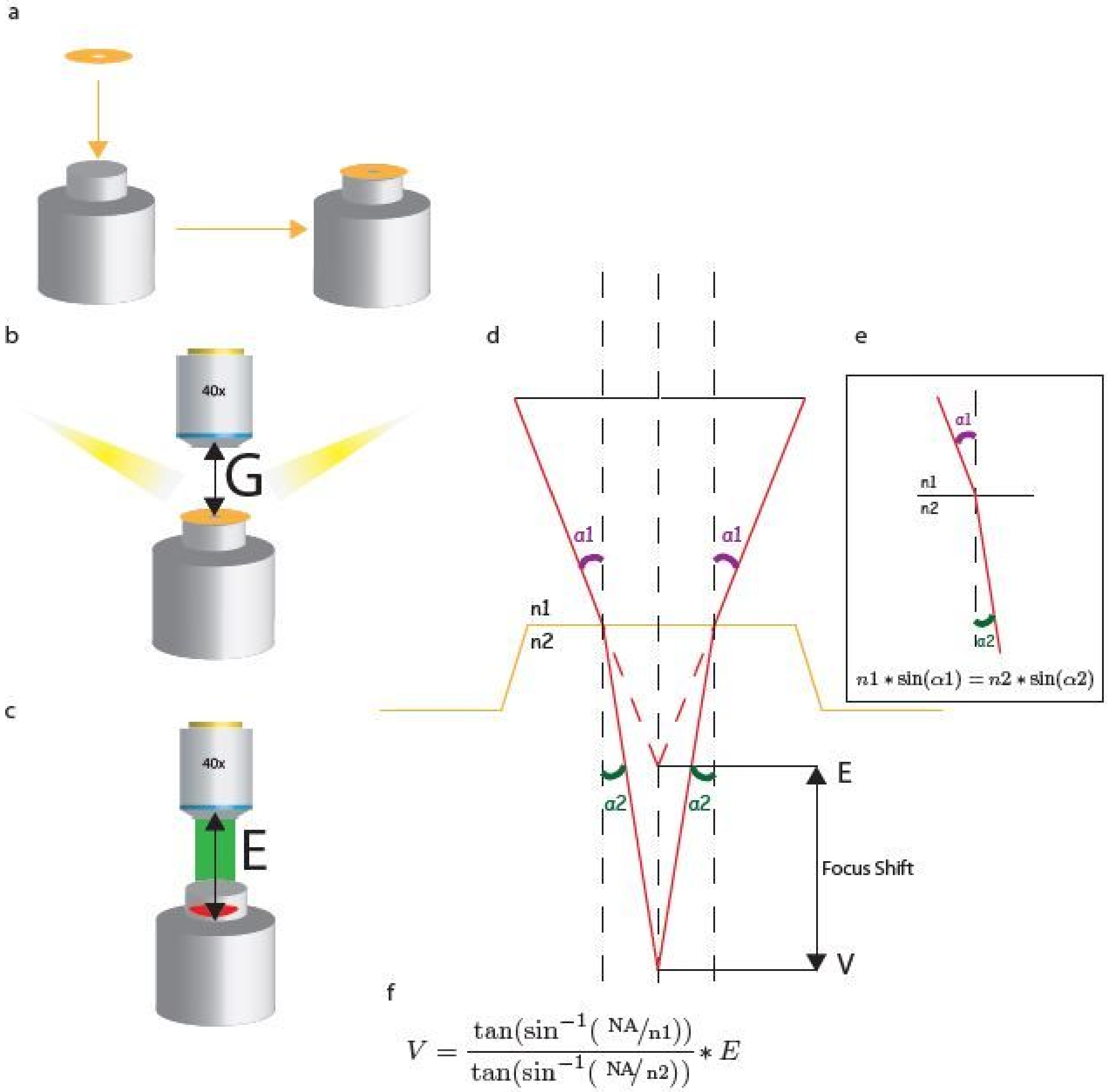

f

$$V = \frac{\tan(\sin^{-1}(\mathrm{NA}/\mathrm{n1}))}{\tan(\sin^{-1}(\mathrm{NA}/\mathrm{n2}))} * E$$

Fig S2 Lemercier et al

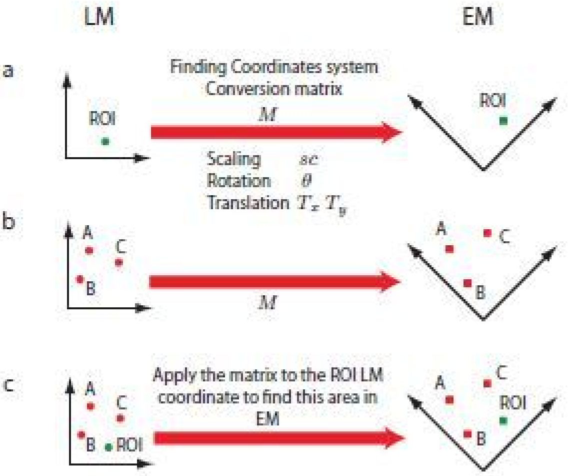

d

$$M * \begin{pmatrix} X_A & X_B & X_C \\ Y_A & Y_B & Y_C \end{pmatrix} = \begin{pmatrix} x_A & x_B & x_C \\ y_A & y_B & y_c \end{pmatrix} \quad (1)$$

$$M = \begin{pmatrix} X_A & X_B & X_C \\ Y_A & Y_B & Y_C \end{pmatrix} * \begin{pmatrix} x_A & x_B & x_C \\ y_A & y_B & y_C \\ 1 & 1 & 1 \end{pmatrix}^{-1} \quad (2)$$

$$\begin{pmatrix} X \\ Y \\ 0 \end{pmatrix} = \begin{pmatrix} sc * \cos(\theta) & sc * (-\sin(\theta)) & T_x \\ sc * \sin(\theta) & sc * \cos(\theta) & T_y \\ 0 & 0 & 1 \end{pmatrix} * \begin{pmatrix} x \\ y \\ 1 \end{pmatrix} \quad (3)$$

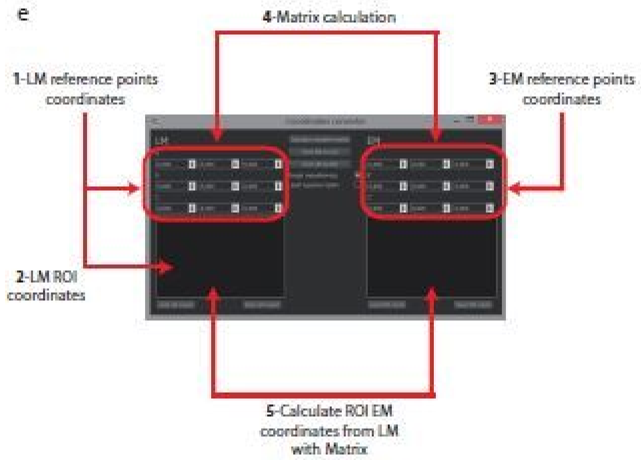

Fig S3 Lemercier et al

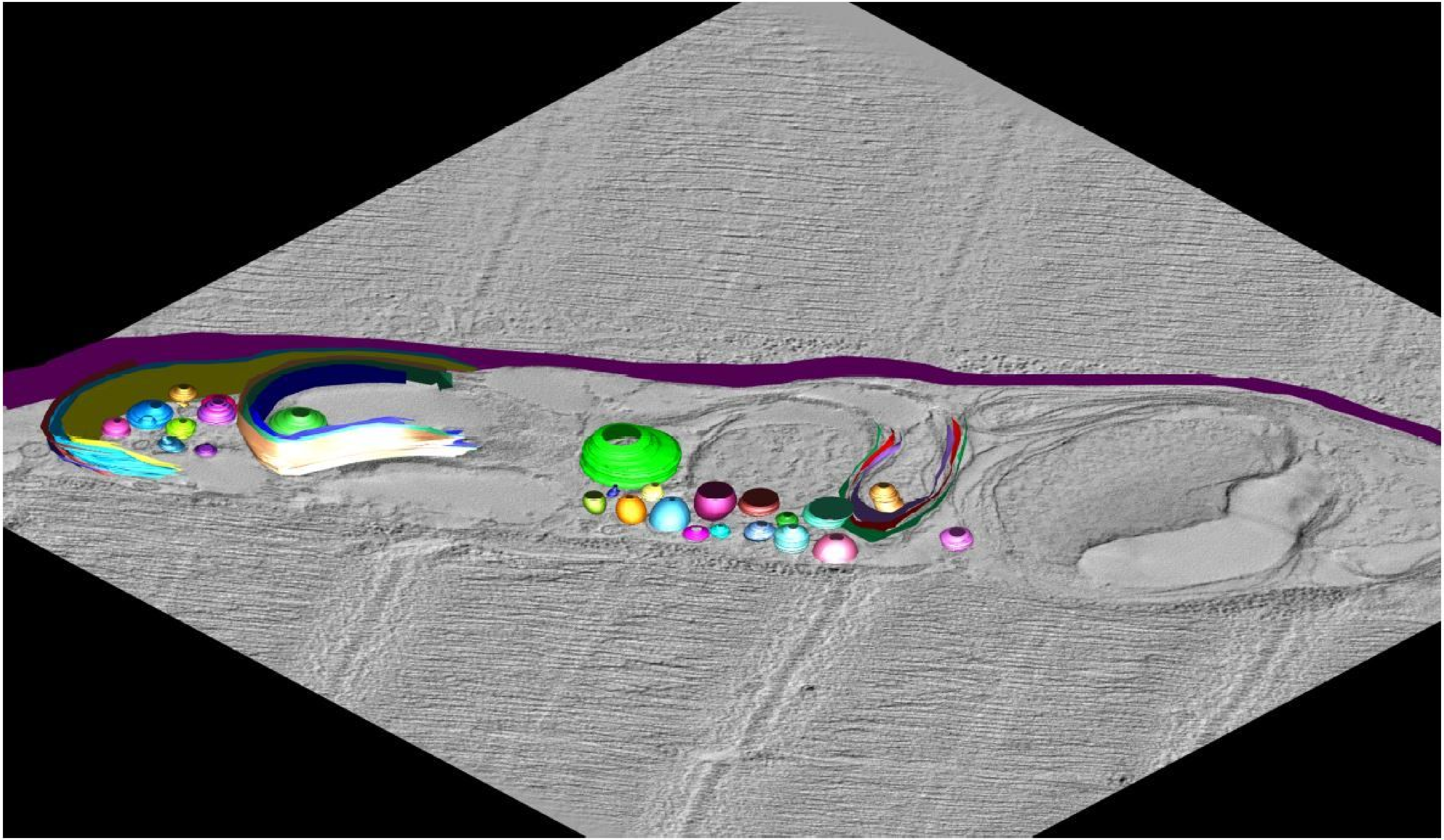

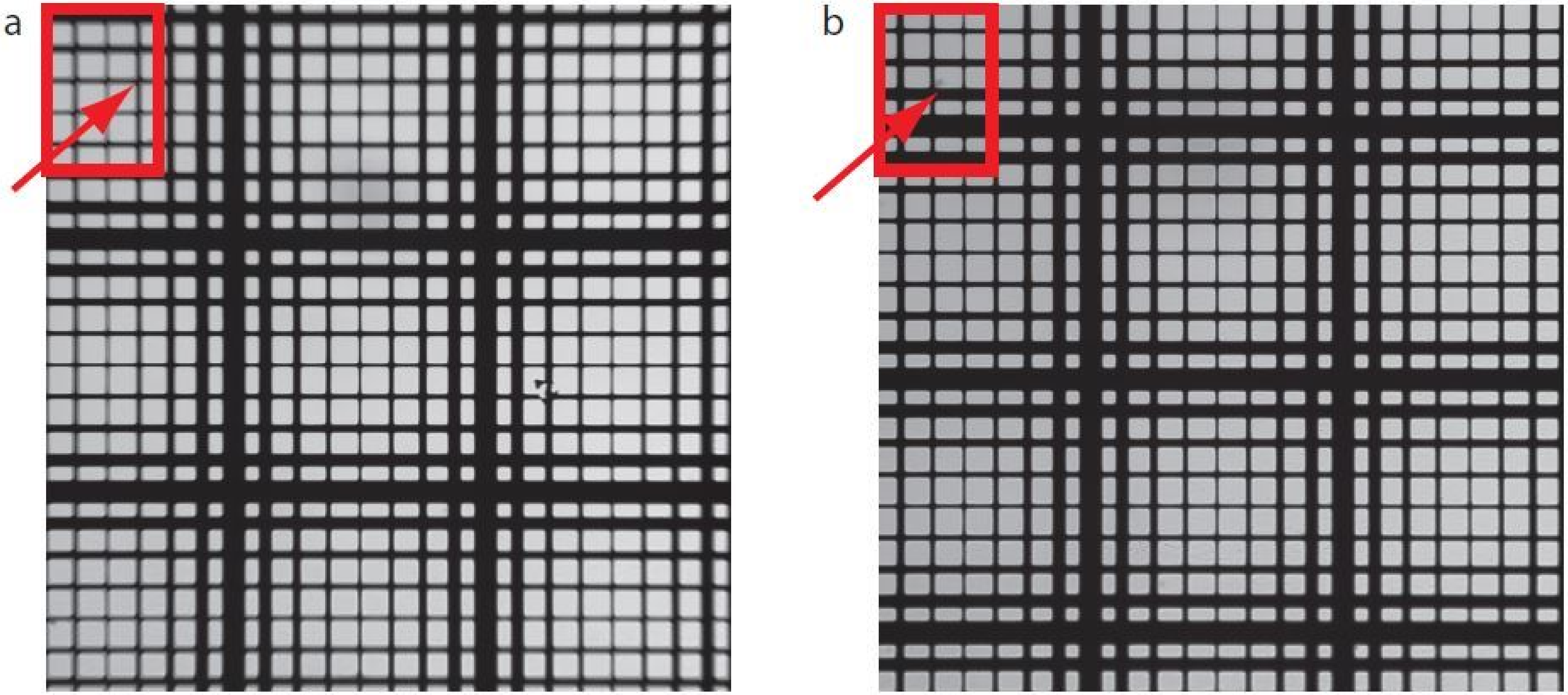

Supplementary Fig: Vignetting
